# Quality Improvement of Biomass Pyrolysis Oil through Esterification

**DOI:** 10.1021/acs.energyfuels.6c01498

**Published:** 2026-08-12

**Authors:** Christian Lindfors, Taina Ohra-aho, Anja Oasmaa

**Affiliations:** 3259 VTT technical research center of Finland Ltd., P.O. Box 1000, Espoo FI-02044 VTT, Finland

## Abstract

Simultaneous esterification and acetalization of bio-oil with ethanol using commercial Amberlyst catalysts at temperatures between 60 and 170 °C were carried out to obtain a less acidic bio-oil with improved stability for small residual boilers. The bio-oil used in the experiments was produced from bark-free pine sawdust, and it was partially dewatered using azeotropic distillation with *n*-heptane before the esterification experiments. A mediocre acidity (carboxylic acid number, CAN 30–50 mg KOH/g) bio-oil was obtained by dewatering and blending the bio-oil with an alcohol. A low-acidity bio-oil (CAN 10–20 mg KOH/g) was obtained with esterification using 50 wt % of ethanol at 80 °C. At higher temperatures (110–170 °C), bio-oil sugars were hydrolyzed and converted into acids, esters, and acetals. The reactions were very dependent on the amount of ethanol. With low concentrations of ethanol (15–20 wt %), dehydration was the main pathway, which also produced water-insoluble humins in larger quantities. Higher concentrations of ethanol reduced the amount of humins and improved the stability of the oil (carbonyl content 0.82 mmol/g) at a short residence time (6 h).

## Graphical abstract

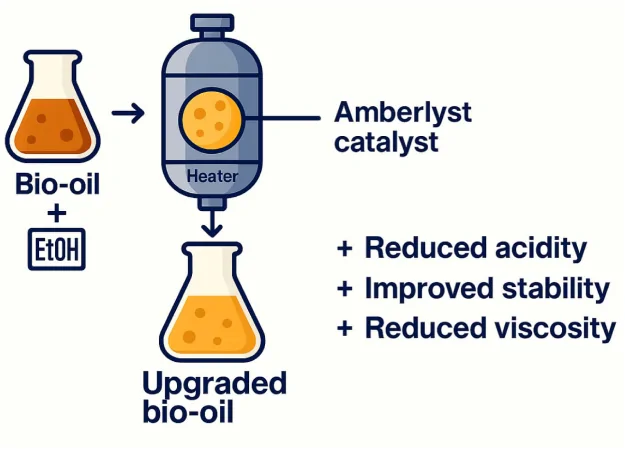

## Introduction

Fast pyrolysis is in its first stage of commercialization, with demonstration plants located in The Netherlands, Sweden, Finland, USA, and Canada.^1^ The first utilization of fast pyrolysis bio-oil (FPBO) has been its use in the heating market to replace fossil heavy and light fuel oil in boilers. The standard EN 16900-2017 specifies the quality requirements and test methods for FPBO in industrial boilers (>1 MW thermal capacity).^2^ Also, the ASTM D7544 standard exists for FPBO use in various types of fuel-burning equipment. For smaller-scale boilers (20 to 200 kW), used in residential heating and diesel engine applications, more information on bio-oil properties and test methods is still needed.

The quality of lignocellulosic bio-oil differs significantly from that of conventional liquid fuels. Bio-oil has a high oxygen content (45–50 wt % wet basis), contains 20–30 wt % of water, and has a heating value (<19 MJ/kg) close to that of biomass. In addition to this, bio-oil is acidic, inhomogeneous due to microchar and extractives, and contains compounds that are not stable during storage and handling.^3–5^ All these properties have a great impact on storage, transport, and combustion technology in terms of the injection system, burner design, flame stability, emissions, and material compatibility.^6^

Alcohol addition has been suggested as one of the cheapest methods to improve bio-oil stability and reduce its viscosity.^6–8^ The instability of pyrolysis liquids depends on the organic components in bio-oil, mainly aldehydes, ketones, and sugar-like compounds, which react with each other to form larger molecules. Bio-oil instability is typically observed as an increase in viscosity, a reduction in carbonyl compounds, and an increase in the heavy water-insoluble compounds over time (i.e., “aging”). The aging reactions are enhanced at elevated temperatures, which result in the phase separation of the less hydrophilic fraction. If the temperature is increased to >200 °C, the separated viscous oil polymerizes into gummy-like and finally char-like material.^4,8–11^

By the addition of an acid catalyst to the alcohol and bio-oil mixture, esterification of the acids and acetalization of the aldehydes, ketones, and sugars take place and a bio-oil with lower acidity, reduced polarity, improved stability, and higher energy density can be obtained (Scheme 1).^12–14^ The esterified pyrolysis oil is a potential renewable fuel for application in smaller-scale boilers and internal combustion engines either as is or as an additive to reduce CO, NO_*x*_, and soot emissions and to inhibit the formation of the crystal structure of soot.^15,16^

**Scheme 1:**
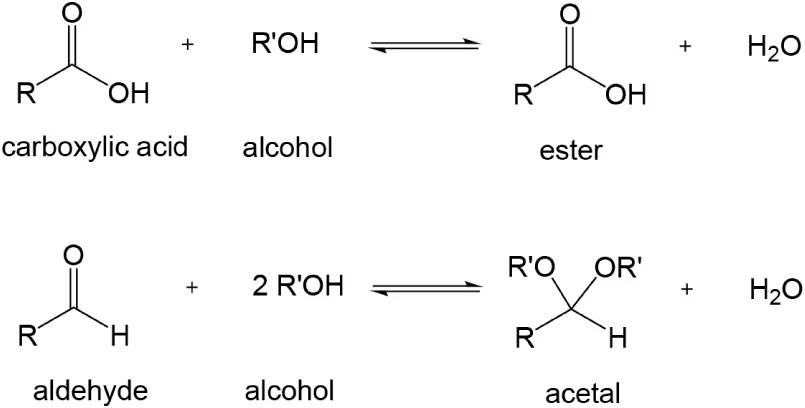
Main Reactions between FPBO Acids and Aldehydes with Alcohols

Esterification of bio-oil refers to an upgrading method, where the aim is to neutralize acidity. It typically includes alcohol addition (30–100 wt %), mild heating (60–120 °C) under a strong acid catalyst (e.g., sulfuric acid), and water removal by distillation. Water removal is essential to achieve complete conversion because water participates in the reverse reactions, which hydrolyze the esters and acetals back to their starting materials.^17^

Two types of studies have been reported to remove water from bio-oil. If esterification is carried out with a water-insoluble alcohol, such as *n*-butanol, water can be removed continuously from the process using azeotropic distillation. In azeotropic distillation, an entrainer solvent (*n*-heptane or petroleum ether) is used to form a low-boiling (70–80 °C) azeotropic mixture with water. The entrainer solvent is insoluble in water and can then be separated and reused in the process. Mahfud et al. at the University of Groningen esterified fast pyrolysis bio-oil while utilizing simultaneous azeotropic water removal with water-insoluble alcohols, such as *n*-butanol and 2-ethylhexyl alcohol. After distillation, the water content decreased to 4.9–8.7 wt % (value of mixture) from the original content of 31.5 wt %. It was a significant decrease but not enough to enhance complete conversion of acids to esters.^18^ If a water-soluble alcohol is used for esterification, water must be removed from the bio-oil before the experiment by azeotropic distillation. Alternatively, molecular sieves can be used to adsorb the water from the liquid. The quantity of molecular sieves has to be large enough to adsorb not only the water in the bio-oil but also the water that results from the condensation reactions.^17,19^

The reactions taking place during esterification are dependent on both the temperature and catalyst loading. Li et al. reported that an increase in the temperature (70–170 °C), reaction time (0–120 min), and catalyst loading (0–6 wt %) resulted in an increase in the conversion of light organic acids and aldehydes to esters and acetals. The reactions were very slow in the absence of an acid catalyst but were non-negligible due to the autocatalytic effects of the organic acids in the raw bio-oil. The formed esters were stable under the selected reaction conditions, but the acetals started to decompose into other compounds as early as 110 °C or during higher catalyst loading (>6 wt %).^12^

Bio-oil contains around 25–35 wt % of sugar-derived compounds, mainly monosaccharides, anhydro sugars (levoglucosan, cellobiosan), and anhydro polysaccharides, which could react under esterification conditions.^20^ The reactions of the sugars in a water-rich and ethanol-rich medium with an acid catalyst are relatively complex and unknown. A simplified reaction scheme for the sugars, developed by Hu et al., is presented in Scheme 2.^21^ During the esterification of acids and the acetalization of aldehydes and ketones, water is also produced, which, in the presence of the acid catalyst, can hydrolyze the sugar oligomers in bio-oil first into levoglucosan at 70–90 °C and after that further into either d-glucose in a water-rich medium or ethyl α-d-glucopyranoside and ethyl β-d-glucopyranoside in an ethanol-rich medium at temperatures of 110–130 °C. Glucose is one of the main precursors for humin (dark-colored carbonaceous and polymeric products) formation in a water-rich medium, which probably results from its reactive functional groups, such as the C1 hydroxyl group. However, if glucose is converted into ethyl α-d-glucopyranoside and ethyl β-d-glucopyranoside, an acetal of glucose, then the reactive C1 hydroxyl group will be protected and the molecule stabilized. Glucose will further form 5-(hydroxymethyl)­furfural (HMF) in a water-rich medium or an ether, 5-(ethoxymethyl)-2-furancarboxaldehyde, in an ethanol-rich medium when the temperature is further increased to around 130–150 °C. The hydroxyl and carbonyl groups in HMF are very reactive in a water-rich medium, and therefore HMF can easily polymerize into water-insoluble humins. Depending on the reaction conditions under which they are produced, humins can appear as solid materials or highly viscous liquids. In an ethanol-rich medium, the hydroxyl group of HMF is converted into an ether group, while the carbonyl group can be converted into an acetal, which stabilizes the molecule. At higher temperatures (150–170 °C), HMF can further produce levulinic and formic acid in a water-rich medium, or ethyl formate and ethyl levulinate in an ethanol-rich medium.^21–27^ This will further result in a more stabilized bio-oil.^22^

**Scheme 2:**
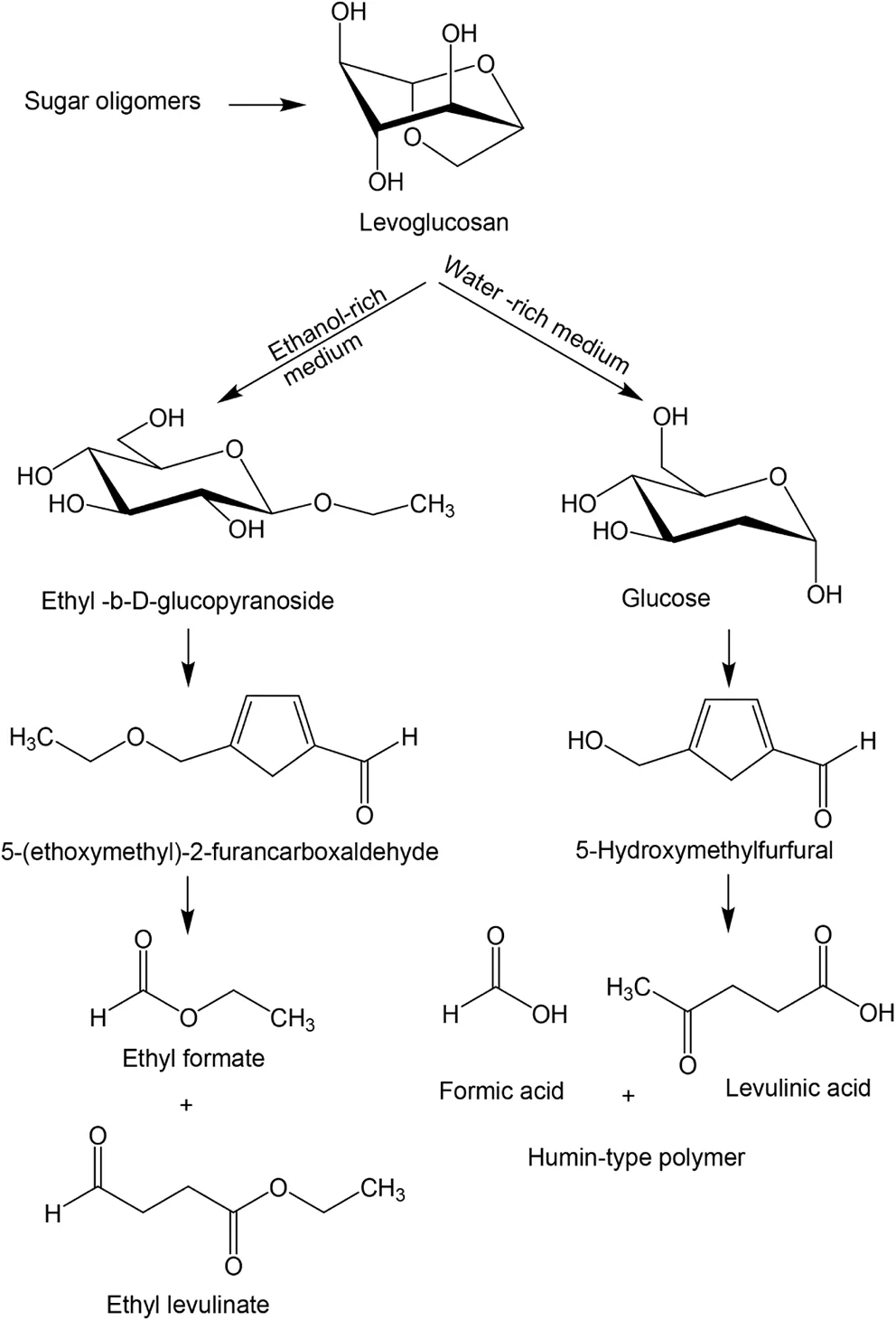
Reaction Pathways of Pyrolysis Sugars in Water-Rich Medium and in Ethanol-Rich Medium^a^

a Reproduced with permission from ref 21. Copyright 2011 Royal Society of Chemistry.

The problem with acid treatment of bio-oil in an aqueous medium is the high formation of water-insoluble humins. The amount of humins decreases usually with a higher alcohol/water ratio.^24,25^ The formation of humins is also dependent on the type of alcohol used in the process. Humins formation according to Wu et al. is independent of the size of alcohol, if monoalcohols are used, but increases if polyalcohols are used.^26^ A higher alcohol concentration is undesirable for the process, since it will increase the cost of the process and decrease the flashpoint of the final product, which changes its safety regulations. Alcohol removal from the esterified products by distillation is also difficult, since the esterified products usually have a boiling point close to that of ethanol or methanol.^27^

The target of the work was to carry out esterification experiments with dewatered bio-oil and ethanol at different alcohol concentrations, catalyst loadings, temperatures, and residence times to obtain a less acidic bio-oil with improved stability for small residual boilers. Lower ethanol concentrations (15–20 wt %) were also tested under optimal esterification conditions to have a moderate alcohol consumption, with a limited increase in the flash point of the stabilized bio-oil.

## Experimental Section

### Bio-Oil Dewatering by Azeotropic Distillation

Esterification experiments were carried out using bio-oil (Table 2), which was produced from bark-free pine sawdust in the BTG (NL) pyrolysis pilot plant. Before esterification, bio-oil was partially dewatered using azeotropic distillation with *n*-heptane (purity ≥ 99%, Sigma-Aldrich) as the entrainer solvent. Azeotropic distillation was carried out using a Dean–Stark apparatus at 90 °C for 3 h. In the method, FPBO (250 g) and *n*-heptane (25–50 g) were weighed in a 500 mL three-neck flask. The flask was heated using an oil bath and magnetically stirred during the distillation. As the distillation was carried out, the distillate was collected in the uninsulated liquid trap (Figure 1). The distillate separated into two distinct liquid phases: the organic solvent phase (*n*-heptane) on the top and the aqueous phase on the bottom. The aqueous phase was periodically removed via a three-way cock, while the solvent phase was allowed to continuously flow back into the distillation flask. After approximately 3 h, the recovery of water had effectively stopped, and the distillation process was halted. Three separate liquid fractions were recovered after this process: the bottom product, i.e., dewatered FPBO, an aqueous distillate, and a separate solvent phase containing the hydrocarbon entrainer.

**Figure 1:**
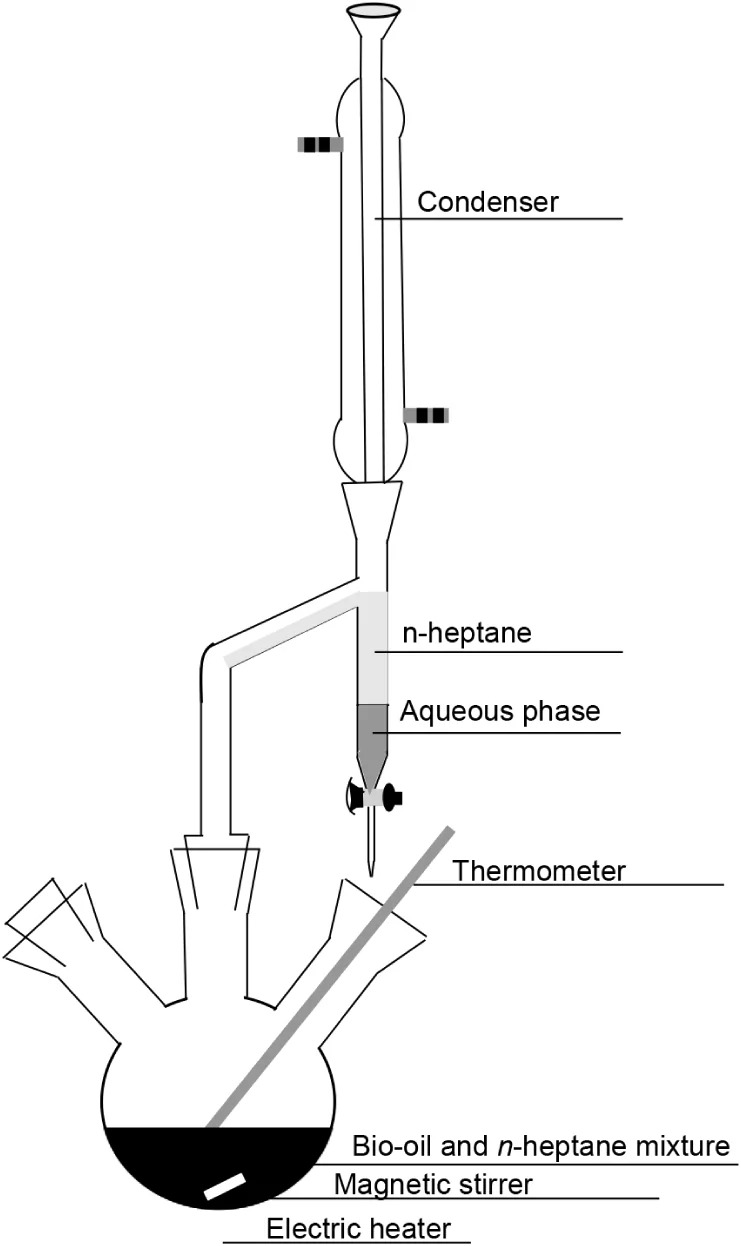
Overall arrangement of the Dean–Stark apparatus.

### Extraction of the Sugar Fraction from FPBO

FPBO contains around 25–35 wt % of sugar-derived compounds, mainly monosaccharides, anhydro sugars (levoglucosan, cellobiosan), and anhydro polysaccharides, which can react with alcohol during esterification at higher temperatures (70–170 °C).^20,21^ To better understand the reactivity of the sugars, a few esterification experiments were carried out with the sugar fraction separated from bio-oil. The sugar fraction was prepared by extracting the bio-oil first with water (1:3) into a water-soluble and water-insoluble fraction (Figure 2). The water-soluble fraction was further extracted with diethyl ether to remove acids, aldehydes, ketones, and phenols. After ether extraction, the main part of the water was removed from the water-soluble fraction using a rotavapor (≤40 °C).^28^

**Figure 2:**
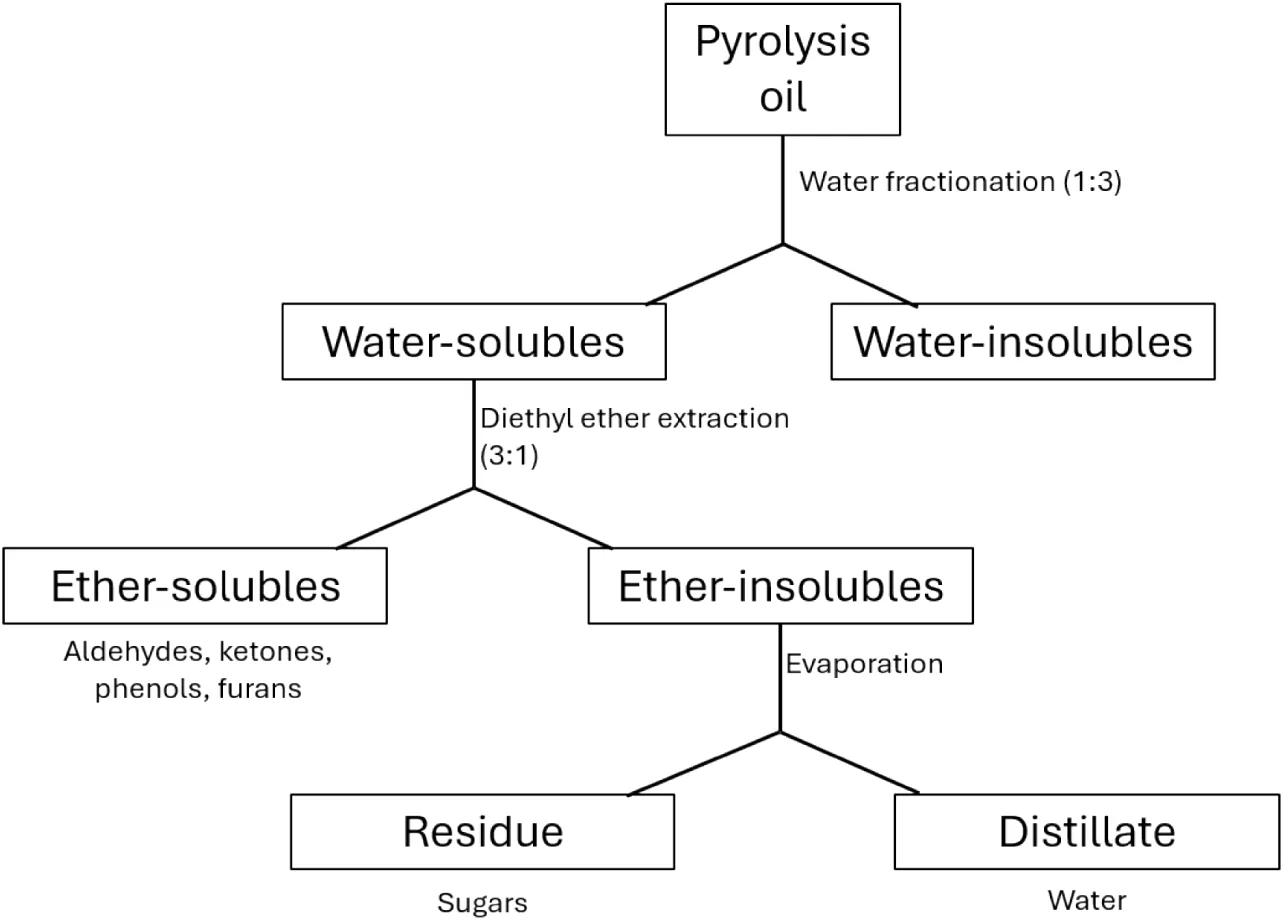
Fractionation scheme for sugars. Reproduced with permission from ref 28. Copyright 2003 American Chemical Society.

### Esterification of Bio-Oil

Esterification experiments were carried out using dewatered bio-oil and ethanol (purity ≥ 99.5%, Sigma-Aldrich). The Dean–Stark apparatus was used for esterification experiments with ethanol at temperatures <80 °C. In the Dean–Stark apparatus, 5 g of catalyst (5 wt % catalyst loading) and 100 g of dewatered bio-oil and ethanol mixture were used. For higher temperature experiments, a tubing bomb setup (40 mL) was used (Figure 3). In the tubing bomb reactor, 2 g of catalyst and 25 g of bio-oil and ethanol mixture were used. The reactor was flushed with nitrogen and pressurized to 15 bar after which the reactor was placed in a sand bath preheated to the desired reactor temperature. Mixing of the solution (160 cycles/min) was carried out by shaking the whole reactor in the sand bath. After the experiment, the whole tubing bomb reactor was lifted into a water bath for quenching. After cooling, the reactor was dried and weighed before opening.

**Figure 3:**
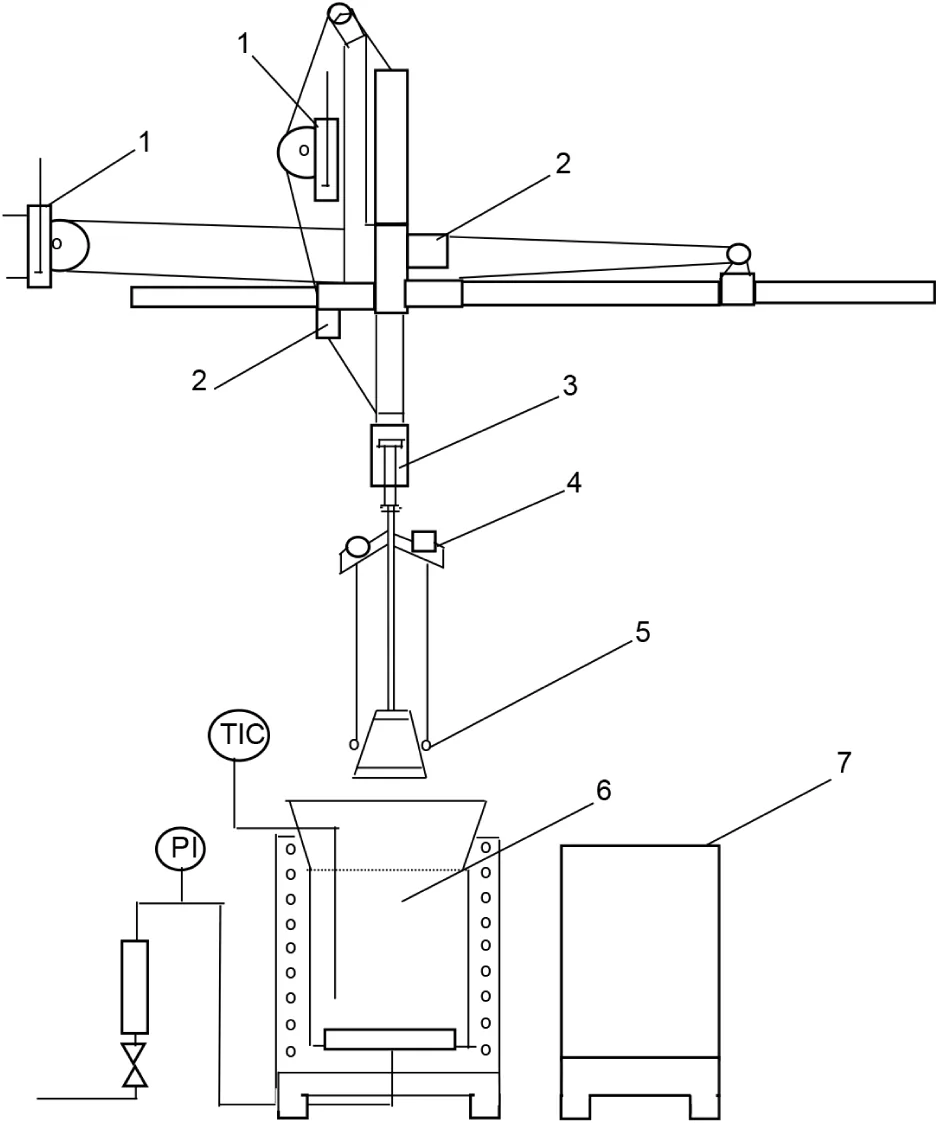
Tubing bomb setup. 1) Air cylinder for transferring the tubing bomb system, 2) stopper, 3) air cylinder for agitation of the tubing bomb, 4) pressure transducer, 5) tubing bomb, 6) fluidized sand bath, 7) water bath.

Solid acid catalysts from ROHM AND HAAS and Dow were used in the experiments (Table 1). The catalyst type was chosen depending on the selected reaction temperature and the catalyst’s maximum temperature tolerance. Catalyst activity and selectivity were not compared. After the experiment, the reaction mixture was filtered to remove the solid catalyst. Fresh catalyst was used in each experiment.

**Table 1:** Catalysts Used for the Esterification Experiments

Catalyst		Amberlyst 15 WET	Amberlyst 36 WET	Amberlyst 45
Supplier		ROHM and HAAS	ROHM AND HAAS	Dow
Physical form		Opaque beds	Opaque beds	Spherical beds
Ionic form as shipped		Hydrogen	Hydrogen	Hydrogen
Concentration of active sites	eg/L	≥1.7	≥1.95	1
	eg/kg	≤4.7	≥5.4	2.95
Uniformity coefficient		≤1.7	≤1.6	≤1.5
Particle mean size	mm	0.6–0.85	0.6–0.85	
Finest content		<0.355 mm: 1% max	<0.425 mm: 0.5% max	<0.355 mm: 0.2% max
Surface area	m2/g	53	33	49
Operation temperature	°C	120	120–150	150–170

The variables changed during the experiments were the ethanol concentration (10–67 wt %), catalyst loading (2–9 wt %), reaction temperatures (60–170 °C), and reaction times (0.5–24 h). Only a single variable was changed at a time; otherwise, the experiments were carried out using a 50 wt % ethanol concentration, 5 wt % catalyst loading compared to the bio-oil ethanol blend, 80 °C reaction temperature, and 24 h reaction time. This reference condition was also repeated to assess the experimental error for the tests.

After these screening tests, experiments were carried out using lower ethanol concentrations (15–20 wt %) at different temperatures. The main targets of these experiments were to reduce the ethanol quantity and to produce a stabilized bio-oil with similar safety regulations compared to FPBO.

The esterified products were mainly analyzed for carboxylic acid number, water content, and ethanol concentrations. To get a better understanding of the reactivity, carboxylic acid number conversion, reaction water, and ethanol consumption were calculated. The carboxylic acid number conversion *X_CAN_* was determined by eq 1. The symbol *m* in the equation represents the mass of the corresponding substance.

1 $$X_{CAN}=100\times \frac{{\left(m_{CAN}\right)}_{in}-{\left(m_{CAN}\right)}_{out}}{{\left(m_{CAN}\right)}_{in}}$$

The reaction water (*Y_water_*) was determined by eq 2.

2 $$Y_{water}=100\times \frac{{\left(m_{water}\right)}_{out}-{\left(m_{water}\right)}_{in}}{{\left(m_{drybio-oil}\right)}_{in}}$$

The ethanol consumption (*Z_EtOH_*) was determined by eq 3.

3 $$Z_{EtOH}=100\times \frac{{\left(m_{EtOH}\right)}_{in}-{\left(m_{EtOH}\right)}_{out}}{{\left(m_{drybio-oil}\right)}_{in}}$$

### Liquid Analyses

The bio-oil used in the experiments was analyzed for physical and chemical properties before and after esterification. Water content was analyzed by Karl Fischer titration using a Metrohm 795 KFT Titrino titrator (ASTM E 203). Elemental composition analysis (CHN) was carried out using an Elementar VARIOMAX CHN analyzer (ASTM D 5291) and a higher heating value (HHV) was determined using an IKA Werke C 5000 Control calorimeter (DIN 51900). Carboxylic acid number (CAN) was determined with a 785 DMP Titrino analyzer (ASTM D 664), and micro carbon residue (MCR) was analyzed using an Alcor Micro Carbon Residue Tester (ASTM D 4530). Carbonyls were analyzed by titration. The method uses the reaction between hydroxylamine hydrochloride and pyridine to determine more than 30 aliphatic, alicyclic, and aromatic aldehydes and ketones. The purpose of pyridine in the reaction is to ensure complete oxidation. The acid released in the form of pyridinium hydrochloride is determined by titration. From the amount of acid, the number of carbonyl groups in the sample can be obtained. Carboxylic acids or esters do not react.^11^ The formation of humins was determined using the solvent fractionation scheme as water-insoluble material. In this method, bio-oil is extracted with water (1:10) into a water-soluble (WS) and a water-insoluble (WIS) fraction. The amount of the water-insoluble fraction was weighed, and its mass fraction was calculated. Besides the above-mentioned methods, two gas chromatography-based characterization methods were also employed. In the first GC method, bio-oil was extracted by water, and the water-soluble compounds were quantitatively analyzed using an Agilent Technologies 7890A gas chromatograph. The GC was equipped with an Agilent 19091 N-136 HP-Innowax column, which had a length of 60 m and an inner diameter of 250 μm. Helium was used as the carrier gas. A flame ionization detector was used at a temperature of 280 °C for detecting the quantifiable compounds. The temperature program for the column oven consisted of first heating the column to 60 °C using a temperature ramp of 3 °C/min. This was followed by heating the column to 230 °C over a time period of 30 min. Calibration was carried out using 40 water-soluble model compounds, and *n*-butanol was used as the internal standard. The list of quantified compounds can be seen in ref 5. A more detailed analysis of the esterified sugar fraction was carried out with an Agilent 7890A GC equipped with an Agilent 5975C inert MSD and a DB-5 MS capillary column (30 m × 0.25 mm, film thickness 0.25 μm) as their trimethylsilyl derivatives. Compound detection with a mass scan range of *m*/*z* between 40 and 800 (EI 70 eV) was used. The MS identifications were based on the use of a commercial Wiley database and in-house spectra collections. For GC/MS, 500-μL samples containing xylitol (10 μL of 20 g/L solution) as the internal standard were evaporated to dryness, dissolved in pyridine, and trimethylsilylated with 0.2 mL of N,O-bis­(trimethylsilyl) trifluoroacetamide, containing 10% trimethylchlorosilane.

## Results

### Chemical Composition of the Dewatered FPBO and Sugar Fraction

The chemical compositions of the starting and dewatered FPBO and the sugar fractions used for the experiments are presented in Table 2.

**Table 2:** Chemical Composition of Bio-Oil before and after Dewatering and Sugar Fractions^a^

Oil sample	Method	Unit	Bio-oil from clean wood	Bio-oil after dewatering	Sugar fraction
Water, wt %	ASTM E 203	wt %	19.0 ± 0.5	1.2 ± 0.5	5.5 ± 0.5
		wt %			
Carbon, wt %	ASTM D 5291	wt %	45.6 ± 0.7	55.9 ± 0.7	46.9 ± 0.7
Hydrogen, wt %	ASTM D 5291	wt %	7.4 ± 0.3	7.7 ± 0.3	6.6 ± 0.3
Nitrogen, wt %	ASTM D 5291	wt %	0.1 ± 0.03	0.1 ± 0.03	0.2 ± 0.03
MCR, wt %	ASTM D 4530	wt %	19.8 ± 1	28.7 ± 1	26.5 ± 1
CAN, mg KOH/g	ASTM D 664	mg KOH/g	64 ± 6	46 ± 6	42 ± 6
HHV, MJ/kg	DIN51900	MJ/kg	19.3 ± 0.1	23.9 ± 0.1	18.7 ± 0.1
LHV, MJ/kg	DIN 51900	MJ/kg	17.6 ± 0.1	22.2 ± 0.1	17.2 ± 0.1
Carbonyl content, mmol/g		mmol/g	4.5 ± 0.2	4.0 ± 0.2	4.7 ± 0.2
Viscosity, cSt	ASTM D 445	cSt	31.1 ± 0.2	7343.0 ± 0.2	-
Density, kg/l	ASTM D 4052	kg/l	1.181 ± 0.001	1.257 ± 0.001	-
C dry, wt %		wt %	56.3 ± 0.7	56.6 ± 0.7	49.6 ± 0.7
H dry, wt %		wt %	6.5 ± 0.2	7.7 ± 0.2	6.3 ± 0.2
N dry, wt %		wt %	0.1 ± 0.03	0.1 ± 0.03	0.2 ± 0.03
O dry (by difference), wt %		wt %	37 ± 1	36 ± 1	44 ± 1
H/C		mol/mol	1.4 ± 0.5	1.6 ± 0.5	1.5 ± 0.5
O/C		mol/mol	0.5 ± 0.5	0.5 ± 0.5	0.7 ± 0.5
MCR dry, wt %		wt %	24 ± 1	29 ± 1	28 ± 1
CAN db, mg KOH/g		mg KOH/g	79 ± 6	46 ± 6	45 ± 6
HHV dry, MJ/kg		MJ/kg	23.8 ± 0.1	24.2 ± 0.1	19.7 ± 0.1
LHV dry, MJ/kg		MJ/kg	22.4 ± 0.1	22.5 ± 0.1	18.4 ± 0.1

a Standard deviation for each method is reported in the table.

During azeotropic distillation, around 95 wt % of the water present in the original FPBO was removed. In addition to this, approximately 8 wt % of the organics were lost to the aqueous phase. These organics were mainly light volatile compounds, which lowered the acidity and increased the carbonyl and oxygen content of the bio-oil. The removal of these compounds according to Oasmaa et al. have a positive impact on the storage stability of FPBO.^29^ In addition to this, less alcohol is needed for the esterification and acetalization of the compounds in pyrolysis oil. The micro carbon residue exhibited a clear increase after dewatering. This is partly due to the removal of water and volatile organics, but it is also possible that some polymerization reactions took place during the dewatering process. The viscosity of the FPBO also underwent a significant increase. As can be seen in Table 2, the dewatered FPBO was practically solid at 40 °C. Ethanol blending can remedy this situation, but handling the dewatered FPBO is, nevertheless, clearly more challenging compared to the original freely flowing liquid.

The sugar fraction separated from the bio-oil is a syrup-like material with a higher oxygen content compared to the starting bio-oil. The water content of the sugar fraction was higher compared to that of the bio-oil after dewatering, which might affect its reactivity during esterification.

## Esterification of Bio-Oil

### Effects of Catalyst Loading and Ethanol Concentration on Reactivity during Esterification

Esterification experiments were started by testing the effect of catalyst loading (2–9 wt %) and ethanol concentration (10–67 wt %) on the reactivity of the acids in the bio-oil. The influence of catalyst loading was monitored by measuring the acidity and water content of the esterified product and calculating the amount of water produced during the reaction (Figure 4). At higher catalyst loading, reaction water increased, and the concentration of acidic constituents decreased, due to more catalyst active sites. The changes in acidity were still relatively small within the 4–9% catalyst loading. Khuenkaeo et al. tested also the effect of catalyst loading and ratio of bio-oil to external alcohol and found that at a 1.5% Amberlyst-15 catalyst loading the ester formation decreased.^30^ This, according to Liu et al. depend on the fact that mass transfer is high, and the additional amount of catalyst cannot influence the process so greatly anymore.^31^ Similar behavior for aldehydes and ketones with increased amounts of catalyst was observed by Li et al.^12^ The higher amount of water, on the other hand, indicates that there are also other compounds than acids, aldehydes, and ketones which compete for the same catalyst acidic sites such as sugars, furans, and phenolics.^32^

**Figure 4:**
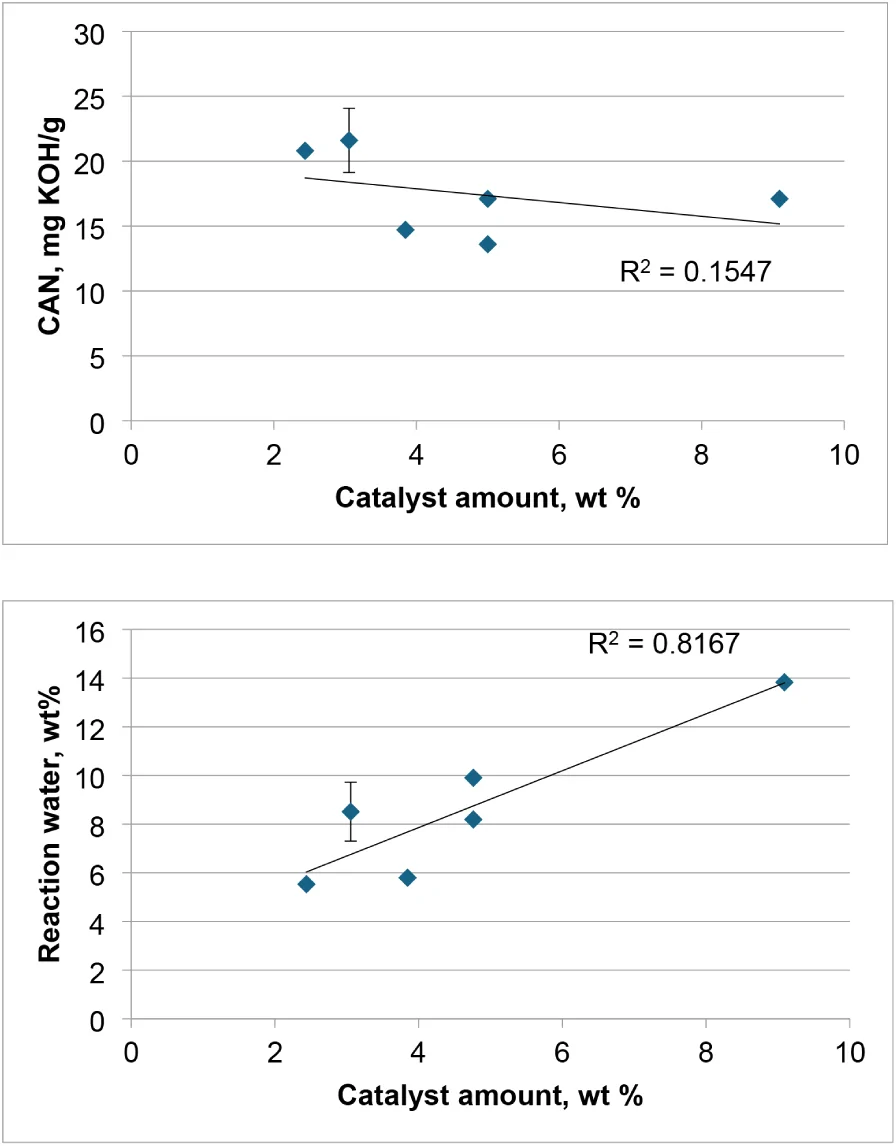
Influence of catalyst loading (2–9 wt %) on acidity (top) and reaction water (bottom). Amberlyst 15 catalyst, temperature 80 °C, 50 wt % of EtOH, time 24 h.

The influence of ethanol concentration on the reactivity was monitored by analyzing the acidity, water content, and ethanol concentration in the product and by calculating the reaction water, CAN conversion, and ethanol consumption (Figure 5). Catalytic esterification is a reversible reaction, and thus, the use of an excess of ethanol encourages the reaction to proceed in the forward direction. The experimental results can be compared with the theoretical amount of ethanol needed to complete the conversion. The bio-oil used in the experiments had a CAN number of 45.5 mg of KOH/g. This corresponds to an acid concentration of 45.5/56.11 = 0.8 mol/kg. The carbonyl content, which indicates the amounts of aldehydes and ketones in the bio-oil, was 4 mol/kg. Their neutralization would then need 0.8 + 2 × 4 = 8.8 mol/kg of alcohol, corresponding to 41 wt % of ethanol in the bio-oil feed. A higher ethanol concentration used for esterification resulted in a lower carboxylic acid number in the product liquid. At the highest ethanol concentration (67 wt %), bio-oil acidity was reduced from 45.5 mg KOH/g in the starting oil to around 10 mg KOH/g in the product liquid. Most of the reduction in acidity was caused by the dilution effect of ethanol, which reduced the acidity to a level of 15 mg of KOH/g. To get a better understanding of the conversion of the reactive species in the bio-oil, CAN conversion, reaction water, and ethanol consumption were calculated. Even at the highest ethanol concentration (67 wt %), only 62% of the acid species were converted into esters. Esterification of the carboxylic acids and acetalization of the aldehydes and ketones produce one molecule of water from each compound. The total amount of produced water would then be 0.8 + 4 = 4.8 mol/kg corresponding to 9 wt % of water. This calculation does not consider the fact that aldehydes can occur as hydrates, and thus, during acetalization reactions, two water molecules are released. Our experimental results also show an increase in reaction water and ethanol consumption at higher ethanol concentrations, even if the esterification of the acids was not completed.

**Figure 5:**
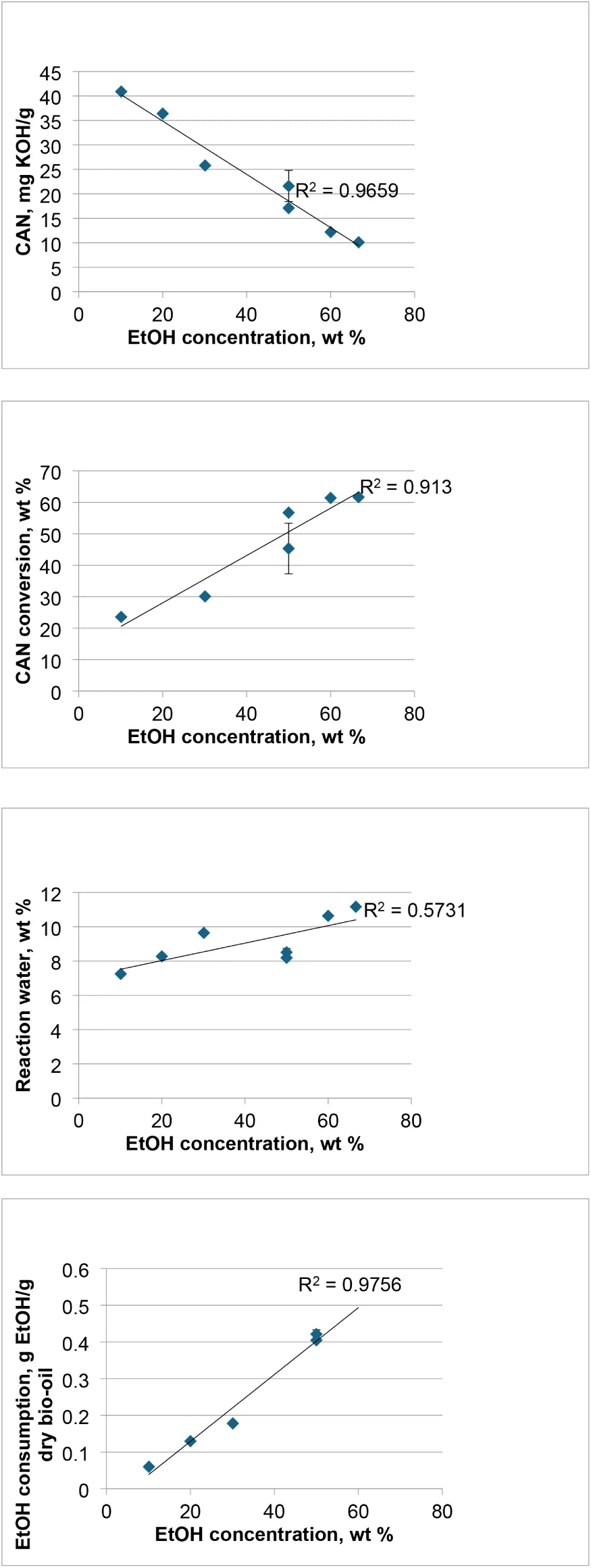
Influence of EtOH amount (10–67 wt %) on acidity, CAN conversion, reaction water, and EtOH consumption. Amberlyst 15, catalyst amount 5 wt %, temperature 80 °C, time 24 h.

The increased water content in the esterified bio-oil will also act as a major inhibitor for further reactions, because esterification is a reversible equilibrium-driven reaction, and thus the excess water drives the reverse hydrolysis reaction of esters back to acids and alcohols. Khuenkaeo et al. compared the esterification of bio-oil with and without water removal and obtained a total acid number (TAN) of 56.95 mg KOH/g from methanol esterification without a water drain. In comparison, water removal during esterification resulted in a significantly lower TAN value for both methanol (14.13 mg KOH/g) and *n*-butanol (16.31 mg KOH/g). Esterification without water removal then appeared to be unable to efficiently convert the high carboxylic acids into esters because of the equilibrium limitation caused by water removal.^30^ The disadvantage of using water removal during esterification with ethanol or methanol is their low boiling point and water solubility, which will result in the accumulation of methanol in the aqueous distillate instead of the bottom product.

### Effects of Reactor Temperature and Residence Time on Reactivity During Esterification

The effect of temperature on the esterification of bio-oil was studied between 60 and 170 °C for 6 and 24 h. In the esterification tests carried out for 6 h, 7 wt % of catalyst was used, while in the 24 h tests 5 wt % of catalyst was used. As expected, temperature had a significant impact on the reactivity. Water formation increased with temperature, while the acidity first decreased and then further increased with temperature (Figure 6). The different catalyst loadings used in the experiments bring one more variable to the series of data points, but according to the previous results, the influence within the 5–7 wt % loading range is relatively small.

**Figure 6:**
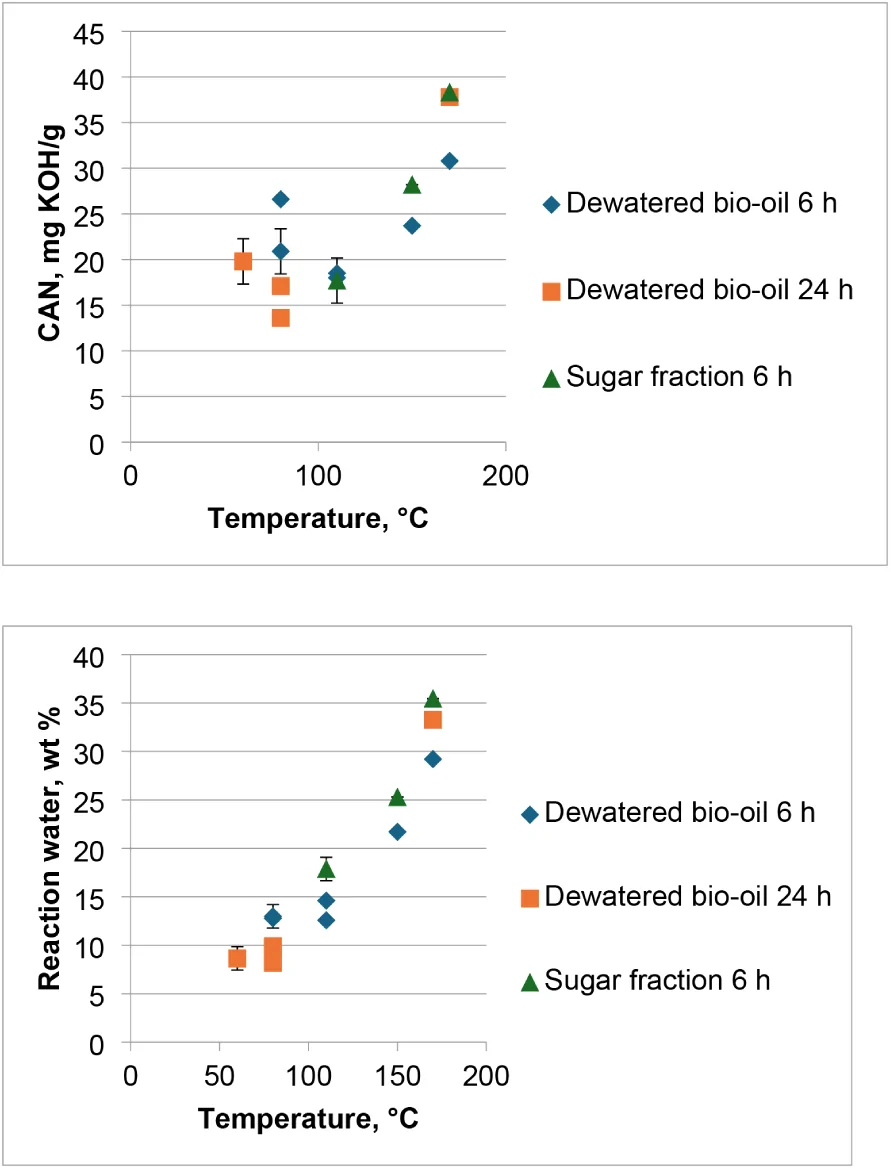
Influence of reactor temperature on acidity and reaction water. Amberlyst 15, 36, and 45, catalyst amount 5 and 7 wt %, 50 wt % EtOH.

The esterification of acids and acetalization of aldehydes and ketones occur according to Li et al. relatively fast^12^ and to confirm this statement, a few experiments were carried out at different residence times while keeping the temperatures at 80 and 170 °C (Figure 7). No significant change in water formation or acidity was seen after a reaction time of 4 h at 80 °C; however, on the other hand, more water and more acids were produced at 170 °C with longer residence times.

**Figure 7:**
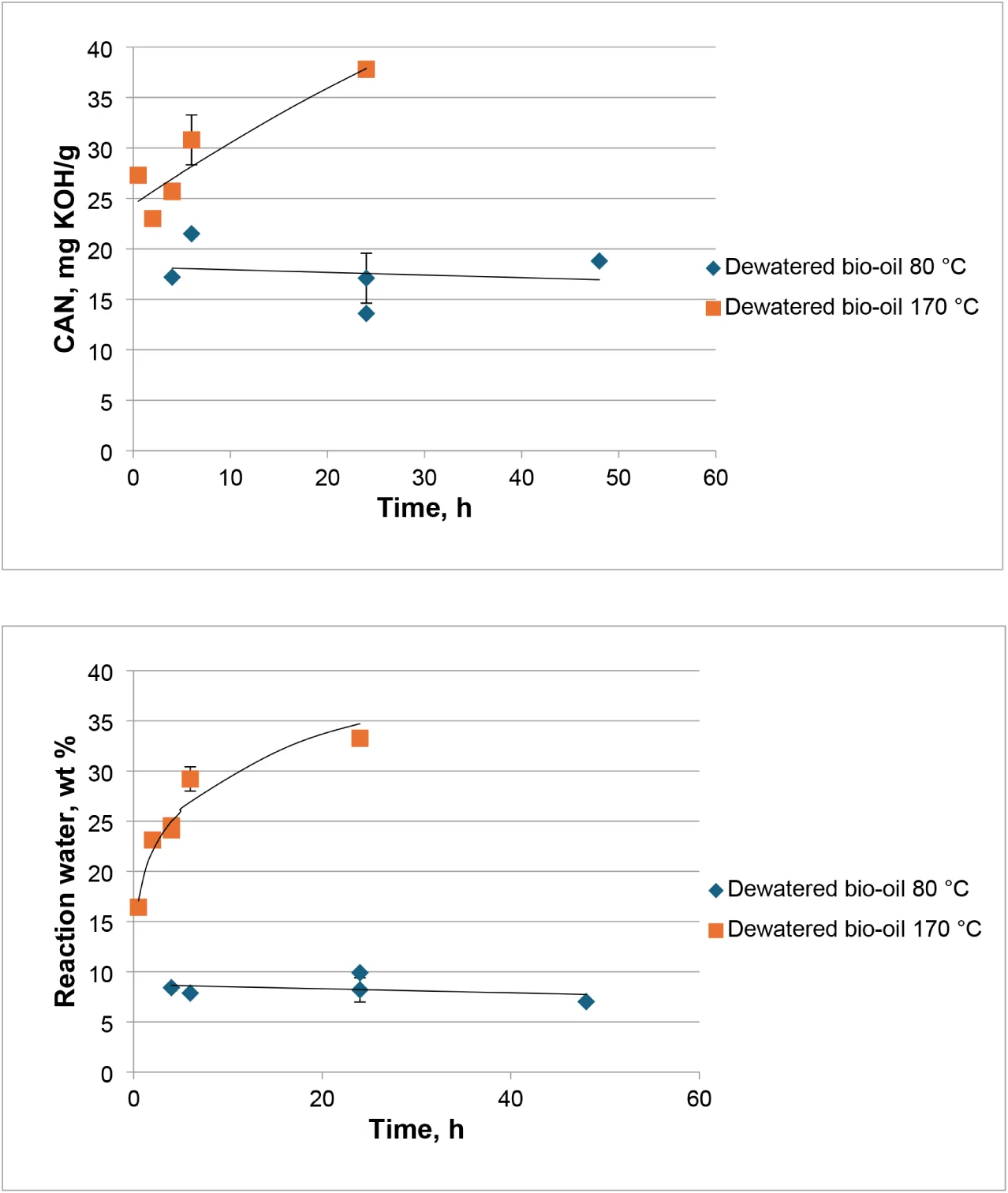
Influence of reaction time on acidity and reaction water. Amberlyst 15, 36, and 45, catalyst amount 5 and 7 wt %, 50 wt % EtOH.

GC analysis of the water-soluble light compounds of bio-oil (Table 3) supports the above-mentioned results. The total amount of aldehydes, ketones, and acids was significantly reduced after esterification at 80 °C for 24 h. On the other hand, an increase in these compound groups was seen after esterification of bio-oil at 170 °C for 24 h compared to the esterification done for a shorter residence time and at a lower temperature with the same residence time. The increase in light compounds at high temperature with the longest residence time indicates that the conversion of bio-oil components is faster than the esterification.

**Table 3:** Concentration of Water-Soluble Compounds in Bio-Oil (wt %)

Bio-oil	Unit	Feed	Treated	Treated	Treated	Treated
EtOH concentration used for esterification	wt %	0	50	50	50	50
Temperature	°C		80	170	170	170
Residence time	h		24	0.5	6	24
Water	wt %	19.0 ± 0.5	7.7 ± 0.5	8.8 ± 0.5	15.1 ± 0.5	17.1 ± 0.5
Aldehydes, ketones	wt %	9.2 ± 0.5	2.2 ± 0.5	1.7 ± 0.5	1.1 ± 0.5	5.8 ± 0.5
Alcohols	wt %	1.2 ± 0.5	31.0 ± 0.5	43.7 ± 0.5	32.6 ± 0.5	19.0 ± 0.5
Ethanol	wt %	0.0 ± 0.5	31.0 ± 0.5	41.7 ± 0.5	29.6 ± 0.5	19.0 ± 0.5
Acids	wt %	7.5 ± 0.5	0.4 ± 0.5	1.3 ± 0.5	1.0 ± 0.5	1.5 ± 0.5
Phenols	wt %	1.0 ± 0.5	0.7 ± 0.5	0.5 ± 0.5	0.3 ± 0.5	0.3 ± 0.5

Bio-oil contains around 25–35 wt % of sugar-derived compounds, mainly monosaccharides, anhydro sugars (levoglucosan, cellobiosan), and anhydro polysaccharides, which could react under esterification conditions.^20^ For comparison, a few tests were carried out with the sugar fraction separated from bio-oil at higher temperatures (Figure 6). As expected, both reaction water and acidity increased when the sugar fraction was treated with the Amberlyst (7 wt %) catalyst and ethanol (50 wt %) at 110–170 °C.

GC/MS results, analyzed from the esterified bio-oil at 170 °C, show also a significant reduction in the concentrations of carbohydrates, mainly levoglucosan, as the residence time was increased from 0.5 to 24 h (Figure 8). A very small concentration of HMF was detected after 0.5 h TOS, but after 24 h TOS, HMF was not detected indicating that it was converted to other compounds. The concentration of levulinic acid, on the other hand, increased already after 0.5 h TOS. This indicated that the carbohydrates were converted into acids instead of esters and acetals. The formation of humins was still small based on visual observation. It is possible that the big carbohydrate molecules in the bio-oil caused some steric hindrance to access the catalytic sites, and therefore the desired esterification and acetalization reactions did not take place.^23^ In the used reactor setup, the FPBO and EtOH mixture was heated very rapidly in the tubing bomb reactor inserted in hot sand to the desired temperature, and therefore the reaction intermediates did not have time to react with the ethanol to be stabilized into acetals and esters. For future tests, stepwise heating could be tested to reduce the dehydration of carbohydrates into acids and increase the esterification and acetalization of FPBO into more stable compounds. Using more severe conditions could also improve the esterification and acetalization reactions. Peng et al. compared the esterification of bio-oil from rice husk with ethanol under conventional, subcritical, and supercritical conditions using HZSM-5 as a catalyst and found that the mass percentage of distillation residue was significantly reduced under supercritical conditions.^33^

**Figure 8:**
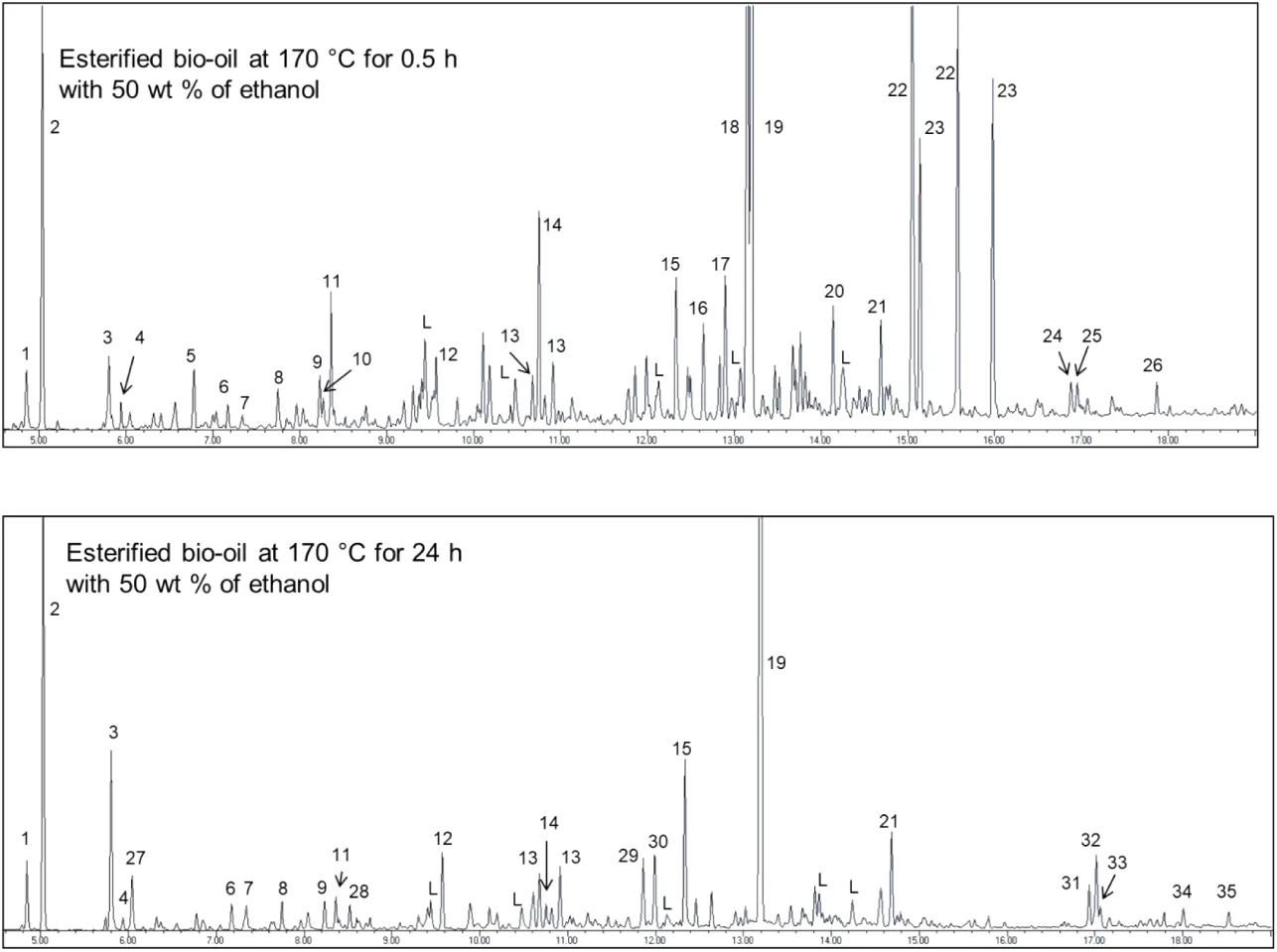
Main compounds identified by GC/MS from the esterified bio-oil at 170 °C for 0.5 and 24 h. 1. Lactic acid, 2. glycolic acid, 3. levulinic acid, 4. 3-hydroxypropanoic acid, 5. 2-hydroxy-3-methyl-2-cyclopentenone, 6. 4-hydroxybutanoic acid, 7. monoethyl succinate, 8. glycerol, 9. succinic acid, 10. 5-hydroxymethylfurfural, 11. catechol, 12. 2,4-dihydroxybutanoic acid, 13. 3-deoxypentonic acid lactones (two peaks), 14. 2,5-dihydroxypentanoic acid, 15. anhydroisosaccharinic acid, 16. galactosan,17. mannosan, 18. levoglucosan, 19. xylitol (int.std.), 20. and 21. unknown carbohydrate derivative, 22. mannose (two peaks), 23. glucose (two peaks), 24–25. unknown hydroxy acids, 26. unknown carbohydrate derivative, 27. 2,5-dihydroxypentanoic acid lactone, 28. glyceric acid, 29–30. unknown polyhydroxy compounds, 31–35. unknowns; L, lignin degradation products.

To determine the quality of the esterified oil, micro carbon residue (MCR) and carbonyl content were measured (Figure 9). The values for the dewatered bio-oil and ethanol mixture were also included in the figures. No significant change in MCR was seen for bio-oil at higher reaction temperatures, while the carbonyl content dropped from 1.28 to 0.82 mmol/g as the temperature was increased from 80 to 170 °C for 6 h. Similar behavior was seen for the sugar fraction. This indicates that a higher temperature selected for esterification will improve the bio-oil’s stability by converting the sugars into more stable compounds. Keeping the bio-oil at 170 °C for too long, on the other hand, is not desirable because of the formation of aldehydes, ketones, and acids, which increased the carbonyl content to a similar level compared to esterification at 80 °C.

**Figure 9:**
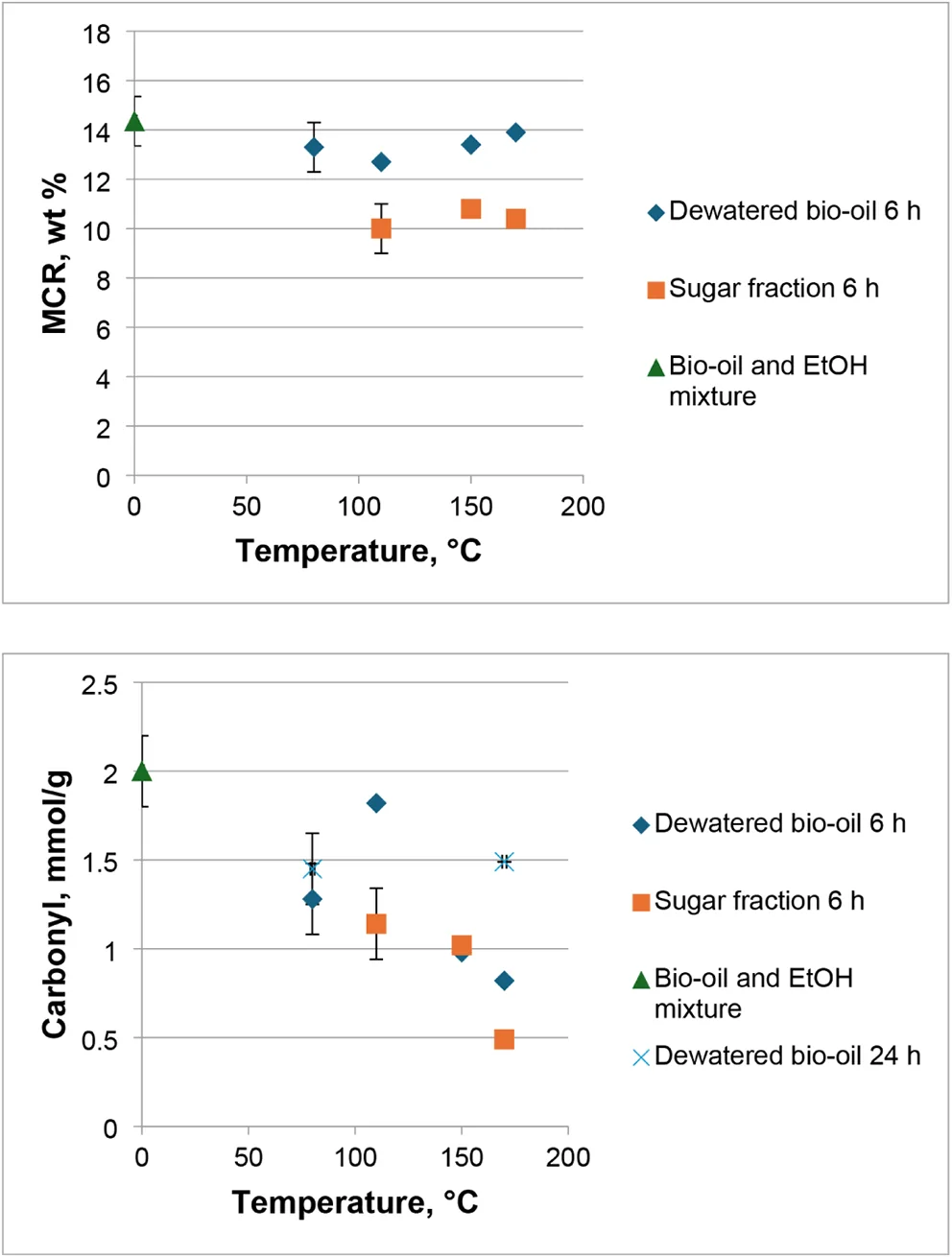
Influence of reaction temperature on bio-oil quality measured as micro carbon residue and carbonyl content.

### Esterification Using Lower Amounts of Ethanol

In the previous esterification experiments carried out, 50 wt % of ethanol was used. This increases both the cost of the process and changes the flashpoint of the product. The flashpoint measured from FPBO was close to 60 °C. This is classified as a Combustible Liquid according to the Globally Harmonized System of Classification and Labeling of Chemicals (GHS). If the flashpoint drops between 23 and 60 °C, the oil will be classified as a flammable liquid (GHS category 3). By increasing the ethanol content of the mixture, the flashpoint drops quite rapidly below 60 °C. To increase the feasibility of esterification, alcohol could either be removed after esterification, or esterification could be carried out with a smaller amount of ethanol. Removal of ethanol from the product by distillation is difficult due to its close boiling points with the esterified product.^27^ Due to the challenges with the isolation of ethanol from the esterified product, esterification experiments were carried out using a smaller amount of ethanol (15–20 wt %). The target was not directly to lower the acidity of the bio-oil, but to make it more stable for further treatment. The reaction water (Figure 10) yield significantly decreased when ethanol concentration was reduced from 50 to 20 wt %, indicating less esterification and acetalization reactions taking place. Instead, sugars were converted into acids. At the same time, polymerization of the sugars took place, which reduced the carbonyl content of the bio-oil but increased the content of water-insoluble material (humins). As a result of the increased amount of water-insoluble humins, oil samples produced with low concentrations of alcohol (25–20 wt %) were very viscous. Using a lower amount of alcohol for high-temperature esterification will then not be beneficial to improve the bio-oil quality for small residual boilers.

**Figure 10:**
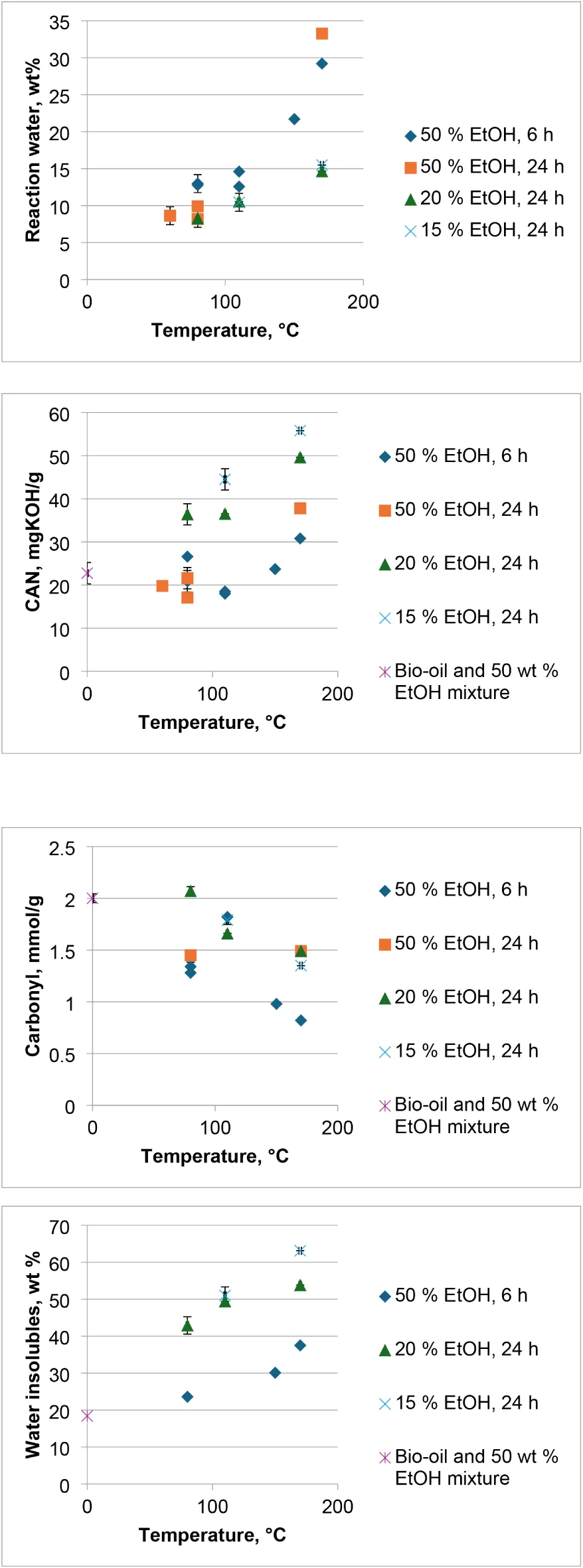
Influence of ethanol concentration and esterification temperature on reaction water, acidity, carbonyl content, and amount of water insolubles. Amberlyst 15, 36, and 45, catalyst amount 5 and 7 wt %.

## Discussion

Despite the reduction in acidity and improvement in oil stability, esterified bio-oil remains inferior to conventional petroleum fuels, which exhibit negligible oxygen content, very low water levels, and minimal acidity, resulting in higher calorific value, lower viscosity, and superior storage stability. Consequently, further upgrading such as hydrodeoxygenation is often required to approach petroleum-like properties. Esterification of bio-oil is fundamentally constrained by its high water content, as the reaction is reversible and governed by equilibrium; excess water shifts the system toward hydrolysis, thereby limiting ester formation and maintaining high acidity. Experimental evidence confirms this limitation, where significantly lower carboxylic acid numbers (CAN) are achieved only when water is actively removed during esterification, highlighting the importance of integrating separation strategies such as azeotropic distillation or reactive distillation to drive conversion. Additionally, a substantial excess of alcohol is often required to drive esterification toward higher conversions. While such approaches improve product quality by reducing acidity, their implementation at an industrial scale introduces additional complexity, requiring efficient reactor–separator integration, robust phase handling, and careful control of mass and heat transfer.

Additional challenges arise from the highly complex and heterogeneous composition of bio-oil, which contains organic acids, phenolics, aldehydes, ketones, sugars, and oligomeric species. These compounds may participate in competing reactions during esterification, leading to polymerization, condensation, and coke precursor formation, which can increase viscosity and negatively affect long-term storage stability. Furthermore, catalyst deactivation remains a significant concern, particularly for heterogeneous acid catalysts, because of pore blockage, carbon deposition, and poisoning by inorganic impurities originating from biomass feedstocks. Mass-transfer limitations associated with the high viscosity and multiphase nature of bio-oils can further restrict the reaction rates and conversion efficiency.

From a feasibility standpoint, esterification is technically mature but strongly dependent on catalyst performance, alcohol selection, and, critically, continuous dehydration; high-boiling alcohols enable effective water removal yet necessitate efficient recovery and recycling systems to maintain process viability. Economically, these requirements translate into increased capital and operating costs due to additional distillation units, energy demand, and solvent management, although these costs may be justified by improvements in acidity, fuel stability, and overall product quality or mitigated through process integration and partial upgrading strategies.

## Conclusions

Alcohol addition combined with catalytic esterification can be used to improve the stability of FPBO and reduce its acidity. A mediocre acidity (CAN 30–50 mg KOH/g) bio-oil was obtained by dewatering and blending the bio-oil with alcohol. A low-acidity bio-oil (CAN 10–20 mg KOH/g) was obtained through esterification using 50 wt % ethanol at 80 °C. Under these conditions, esterification of acids and acetalization of aldehydes and ketones occurred relatively fast, in less than 4 h. At higher temperatures (110–170 °C), sugar oligomers were first hydrolyzed into levoglucosan, which was further dehydrated into acids and acetalized and esterified into more stable compounds. The reactions were very dependent on the amount of ethanol. With low concentrations of ethanol (15–20 wt %), dehydration was the main pathway, which increased the amount of water-insoluble humins. Higher concentrations of ethanol (50 wt %) reduced the amount of humins and improved the stability of the oil (carbonyl content decreased to 0.82 mmol/g) at short residence times (6 h). Keeping the bio-oil at 170 °C for too long (24 h) is, on the other hand, not desirable, because of the formation of aldehydes, ketones, and acids, which increased the carbonyl content to a similar level compared to esterification at 80 °C.

Esterification might be an attractive choice to improve the bio-oil properties in applications where alcohol is the main fuel and bio-oil is added in smaller quantities. Such an application might be in boilers or diesel engines, which are flexible for dual fuels. Further boiler and engine tests are still needed to confirm the application.
